# Disruption of the Thyroid System by the Thyroid-Disrupting Compound Aroclor 1254 in Juvenile Japanese Flounder (*Paralichthys olivaceus*)

**DOI:** 10.1371/journal.pone.0104196

**Published:** 2014-08-04

**Authors:** Yifei Dong, Hua Tian, Wei Wang, Xiaona Zhang, Jinxiang Liu, Shaoguo Ru

**Affiliations:** Marine Life Science College, Ocean University of China, Qingdao, Shandong Province, The People's Republic of China; University of Missouri, United States of America

## Abstract

Polychlorinated biphenyls (PCBs) are a group of persistent organochlorine compounds that have the potential to disrupt the homeostasis of thyroid hormones (THs) in fish, particularly juveniles. In this study, thyroid histology, plasma TH levels, and iodothyronine deiodinase (IDs, including ID_1_, ID_2_, and ID_3_) gene expression patterns were examined in juvenile Japanese flounder (*Paralichthys olivaceus*) following 25- and 50- day waterborne exposure to environmentally relevant concentrations of a commercial PCB mixture, Aroclor 1254 (10, 100, and 1000 ng/L) with two-thirds of the test solutions renewed daily. The results showed that exposure to Aroclor 1254 for 50 d increased follicular cell height, colloid depletion, and hyperplasia. In particular, hypothyroidism, which was induced by the administration of 1000 ng/L Aroclor 1254, significantly decreased plasma TT_4_, TT_3_, and FT_3_ levels. Profiles of the changes in mRNA expression levels of IDs were observed in the liver and kidney after 25 and 50 d PCB exposure, which might be associated with a reduction in plasma THs levels. The expression level of ID_2_ mRNA in the liver exhibited a dose-dependent increase, indicating that this ID isotype might serve as sensitive and stable indicator for thyroid-disrupting chemical (TDC) exposure. Overall, our study confirmed that environmentally relevant concentrations of Aroclor 1254 cause significant thyroid disruption, with juvenile Japanese flounder being suitable candidates for use in TDC studies.

## Introduction

Polychlorinated biphenyls (PCBs) have been listed as one of 21 persistent organic pollutants (POPs) under the Stockholm Convention, due to their recalcitrance to degradation and tendency to biomagnify up the food chain. PCBs are widely studied TDCs that potentially cause various abnormalities in the thyroid system of vertebrates [1]–[3], especially in amphibians and mammals [4]. Recently, the disturbance of fish thyroid systems by PCBs has received increasing research focus; however, the thyroidal responses of fish to PCBs has shown variable results in different studies [5]. For example, Aroclor 1242 and 1254 (commercial PCBs mixtures) lowered plasma 3,5,3′ -triiodothyronine (T_3_) levels without altering plasma thyroxine (T_4_) levels when fed to adult coho salmon (*Oncorhynchus kisutch*) [6]. The injection of Aroclor 1254 has been shown to increase plasma T_3_ levels and delay the plasma T_4_ surge commonly associated with smoltification [7]. These variable effects on thyroid hormone (THs) levels may be related to the physiological stage or age of fish used in different laboratory studies. Most of these studies preferentially used adult fish of sufficient size/age to either obtain adequate blood samples for THs measurement or the assessment of other thyroid indices, while only a few studies have used juvenile fish to assess the thyroid disrupting effects of PCBs [5].

Some researchers recommended that young developing fish should be the focus of future studies on thyroid disruption, because juvenile fish are more dependent on the regulation of THs and more sensitive to TDCs compared to adult fish [5], [8]. THs have been linked to a multitude of important functions in early development of fish, such as growth, tissue differentiation, and metamorphosis [8], [9]. In Japanese flounder (*Paralichthys olivaceus*), the exogenous administration of THs or elevation of endogenous T_4_ levels by thyroid stimulating hormone (TSH) induces advanced metamorphosis, while thiourea (TU, an anti-thyroid drug) treatment delays the metamorphosis process [10], [11]. Exogenous THs also induce the transition of muscle proteins, replacement of erythrocytes, skin pigmentation, and development of the gastric glands in fish [12], [13]. These findings indicate that THs are fundamental for the early development and growth of fish, and that TH disruption in juvenile fish may cause growth retardation or abnormal development. Therefore, juvenile fish are assumed to be particularly susceptible to thyroid disruption.

A series of endpoints have been proposed to assess the effects of PCBs on the fish thyroid cascade, and mainly include central controlled effects and peripheral controlled effects [8]. Measurement of the central control of the thyroid cascade may be accomplished *via* thyroid histopathological analysis, in addition to measurement of plasma total and free THs levels [14]–[18]. The peripheral control of the conversion of T_4_ to T_3_ may be assessed *via* a suite of iodothyronine deiodinase (IDs) activities in the liver or other extra-thyroid tissues [19], [20]. Three ID isotypes are mainly expressed in teleosts, with these enzymes presenting different catalytic properties [21]. In particular, ID_1_ exhibits both outer ring-deiodination (ORD) and inner ring-deiodination (IRD) activities; however, when combined with its preferred substrate, 3,3′,5′-triiodo-l-thyronine (rT_3_), this enzyme is considered to become even more involved in the degradation of THs, particularly the inactivation of rT_3_ to 3,3′ -diiodo-l-thyronine (3,3′ -T_2_). ID_2_ activates the ORD pathway, by converting T_4_ into T_3_. ID_3_ catalyses the IRD pathway, which converts T_4_ and T_3_ into the inactive metabolites rT_3_ and 3,3′-T_2_, respectively [22]–[24].

The Japanese flounder is an economically important species that is considered to be an ideal model organism for the study of thyroid disruption. The important roles of THs during the early stage of the development of this flatfish have been extensively demonstrated, particularly during metamorphosis [25]–[28]. To date, effects of PCBs on the thyroid system of the Japanese flounder remain unclear. This study aimed to obtain an integrated insight into the effects of environmentally relevant concentrations of Aroclor 1254 on the thyroid system of juvenile Japanese flounder. Changes in the development and growth of this fish species were examined, and the tissue levels of PCB congeners were measured. We anticipate that these analyses will indicate the potential suitability of using juvenile Japanese flounder as candidates for use in TDC studies.

## Materials and Methods

### Ethics statement

The fish were handled according to the National Institute of Health guidelines for the handling and care of experimental animals. The animal utilization protocol was approved by the Institutional Animal Care and Use Committee of the Ocean University of China. All surgery was performed under MS-222 anesthesia, and all efforts were made to minimize suffering.

### Animals

Experimental trials were conducted in the marine life science college of Ocean University of China. A total of 360 juvenile Japanese flounder (80 days post hatching) were purchased from a commercial fish farm in China. The fish were raised in 240-L tanks containing 200 L of sand-filtered natural seawater (pH 8.0±0.1; 33 ppt salinity) at an ambient temperature (23±3°C). To minimize the aggressive behavior of juvenile fish, a 24-h dark photoperiod (light/dark cycle, 0/24 h) was maintained, with the tanks only being lit up 10 min before each feeding. Fish were fed a commercial flounder feed (Marubeni Nisshin feed, Chuo-Ku, Japan) 4 times a day (2% total fish weight per tank per day) between 08:00 and 20:00. Fish were allowed to acclimate to experimental conditions for 2 weeks prior to the initiation of experiments. The average wet body weight (W_T_) of the fish used in the experiment was 6.21±1.77 g, and the total body length (L_T_) was 8.04±1.54 cm.

### Experimental design and fish sampling

Fish were randomly assigned to a control group and 3 treatment groups (size of each group *n* = 90 in each case). Juvenile Japanese flounder were exposed to Aroclor 1254 (AccuStandard Inc, NH, USA, CAS 11097-69-1) at 0 (control), 10, 100, and 1000 ng/L. Aroclor 1254 stock concentrate (1 mg/mL) was made up in ethanol (50 mg Aroclor 1254 was dissolved in 50 mL ethanol). During exposure, two-thirds of the test solutions were changed once per day, and the appropriate amount of seawater and stock solution was added to maintain the specified chemical concentrations.

Fish were deprived of food on the last day of exposure. After 25 and 50 days of exposure, all fish were anesthetised in MS-222 (Sigma, St. Louis, MO, USA), and rinsed with distilled water. The L_T_ and W_T_ of the fish in each tank (*n* = 9) were measured to calculate the condition factor (CF = 100×W_T_ (g)/L_T_ (cm)^3^). Blood was collected in heparinised tubes by puncturing the caudal vein within 3 min of netting the fish. After centrifugation, plasma was collected and stored at −80°C until RIA. In particular, at the 25-day sampling point, the plasma of 2–3 fish was pooled (*n* = 9). The liver and kidney tissues (*n* = 9) were isolated, frozen in liquid nitrogen, and stored at −80°C until further processing. For the histology analysis, the thyroid tissues enclosed in the subpharyngeal area that were sampled at the end of 50 d exposure (*n* = 9) were fixed in formalin fixative for 24 h at 4°C, and stained with hematoxylin-eosin.

### PCB contaminant analysis

The real concentrations for the 7 tracer PCB congeners in whole fish was measured with GC-MS as described in [29]. At the end of 50 d exposure, 2–3 fish (approximately 50 g in total weight) from each group were randomly sampled, lyophilized, and homogenised in 20 g anhydrous sodium sulphate, and were then placed into a Soxhlet extractor. Samples with 7 types of ^13^C recovery internal standards (PCB 28, 52, 101, 118, 153, 138, and 180) were extracted by 350 mL hexane/dichloromethane = 1∶1 (v/v) for 24 h. After primary purification by gel permeation chromatography, the extracts were then placed in acid silica (44% sulphuric acid, w/w) for further purification and component separation. Hexane (20 mL) was used for the complete elution of PCBs. The final eluate was concentrated to 100 µL under nitrogen and then transferred to a GC vial with ^13^C-PCB 202 inlet internal standards. The PCB congeners were analysed using an Agilent 6890N/5973i GC-MS system (Agilent Technologies Inc., Palp Alto, USA). The GC-MS analytical parameters have been conducted by referring to Environmental Protection Series: Reference method for the analysis of polychlorinated biphenyls (EPS 1/RM/31, Canada).

### Thyroid histological processing

All histopathological endpoints were assayed, as described in [16], with minor modifications. Serial sections were examined under a light microscope until 6–14 follicles/fish were found and photographed. Follicular cell height on the pictures was quantified using Image-Pro plus (version 6.0.0.260). Follicular cell height was determined by obtaining 6 measurements at regular intervals along the follicle perimeter (i.e. 36–84 follicular cell height measurements for each fish and 324–756 measurements for each treatment). A grading system was applied for the hyperplasia evaluation: Grade 1, focal hyperplasia; Grade 2, thyroid follicular cell with less than 50% hyperplasia; and Grade 3, thyroid follicular cell with more than 50% hyperplasia. The average score of 9 fish from each treatment was used, which was based on the sum of the grade of each fish. The sum of the number of colloid deletions (per 10 follicles) was calculated (colloid deletion follicle/10 follicles).

### RNA isolation and quantitative RT-PCR

The procedures for RNA extraction and gene expression analysis were performed as previously described by [31]. In brief, total RNA was isolated from the liver and kidney using TRIzol reagent (Invitrogen, Carlsbad, CA, USA) following the manufacturer's instructions. Equal amounts of RNA (1 µg) were reverse-transcribed into cDNA using a PrimeScript RT reagent kit (Takara Bio Inc., Shiga, Japan). Primers were designed for the specific amplification of ID_1_, ID_2_, ID_3_, and 5S-rRNA (an internal control) according to the sequences published in GenBank (Table 1).

**Table 1:** Nucleotide sequences of primers used for real-time PCR and product sizes.

Gene	GenBank Accession No.	Primer sequence (5′–3′)	Amplicon size (bp)
ID_1_	AB362421	GGTGGTGGACGAAATGAATG	147
		TCCAGTAACGAACGCACCTCT	
ID_2_	AB362422	GCACCAGAACTTGGAGGAGAG	142
		GCACACTCGTTCGTTAGACACA	
ID_3_	AB362423	TGGCTGGAGCAGTACAGGAG	103
		TGAGGCAGAATGGGCAGA	
5S-rRNA	AB154836	CCATACCACCCTGAACAC	102
		CGGTCTCCCATCCAAGTA	

All reactions were run on a Eppendorf MasterCycler ep *RealPlex* ^4^ (Eppendorf, Wesseling-Berz-dorf, Germany). Parallel PCR reactions were conducted to amplify the target gene and 5S-rRNA. Real-time PCR was performed in 20 µL reaction mixtures containing 1× SYBR *Premix Ex Taq* (Takara Bio Inc., Shiga, Japan), 0.4 µM for each primer, 0.4 µL of ROX Reference Dye (Takara Bio Inc., Shiga, Japan), and 4 µL of first-strand cDNA (template). The thermal profile was 95°C for 30 s followed by 40 cycles of 95°C for 5 s and 60°C for 30 s. To ensure that a single product was amplified, melting curve analysis was performed on the PCR products at the end of each PCR run. In addition, 2% agarose gel electrophoresis of the PCR products was performed to confirm the presence of single amplicons of the correct predicted size (not shown). 5S-rRNA transcripts were used as housekeeping genes to standardize the results and to eliminate variations in mRNA and cDNA quantity and quality. 5S-rRNA levels were not affected by any of the experimental conditions in the study. The target gene mRNA abundance in each sample, relative to the abundance of 5S-rRNA, was calculated by the formula 2^−ΔΔCt^ and plotted on a logarithmic scale [31].

### Hormone assay

Muscular TT_3_, TT_4_, FT_3_, and FT_4_ concentrations were measured by radio immunoassay (RIA) (Beijing North Institute of Biological Technology, Beijing, China) according to the manufacturer's instructions. The assay detection limits were 0.05 ng/mL for TT_3_, 2 ng/mL for TT_4_, 0.5 fmol/mL for FT_3_, and 1 fmol/mL for FT_4_. The inter- and intra-assay coefficients of variation for all the stated hormones were <10% and <15%, respectively.

### Statistics

All data are presented as the mean ± standard deviation. Data normality was verified using the Kolmogorov-Smirnov test [32], and homogeneity of variance was checked by Levene's test. If the data failed to pass the test, a logarithmic transformation of the data was performed and retested. Significant differences were assessed between each treatment and the control using one-way analysis of variance (ANOVA), followed by Tukey's multiple comparisons test. *P*<0.05 was considered to be statistically significantly different. All statistical tests were conducted using GraphPad PRISM (Version 6.00) software.

## Results

### PCB concentrations in Japanese flounder juvenile

The concentrations of 7 tracer PCB congeners in juvenile Japanese flounder are shown in Table 2. A concentration-dependent bioconcentration of Aroclor 1254 was measured in the whole body of all exposure groups. In the 1000 ng/L treatment, the total concentration of measured PCB congeners (including PCB28, PCB52, PCB101, PCB118, PCB153, PCB138, and PCB180) reached 890.18 ng/g ww.

**Table 2:** The contents of 7 tracer PCB congeners in juvenile Japanese flounder.

Test item	Control	10 ng/L	100 ng/L	1000 ng/L
PCB28	N/D	N/D	N/D	N/D
PCB52	6.90	10.44	28.53	156.15
PCB101	9.21	20.95	52.33	266.03
PCB118	4.04	12.70	40.98	209.51
PCB153	1.14	7.89	18.48	94.39
PCB138	1.77	6.99	30.18	156.30
PCB180	N/D	0.99	2.078	7.78
Total (ng/kg ww)	21.07	59.99	173.48	890.18

N/D: not detected.

### Effects of Aroclor 1254 on the growth of juvenile Japanese flounder

**Figure 1:**
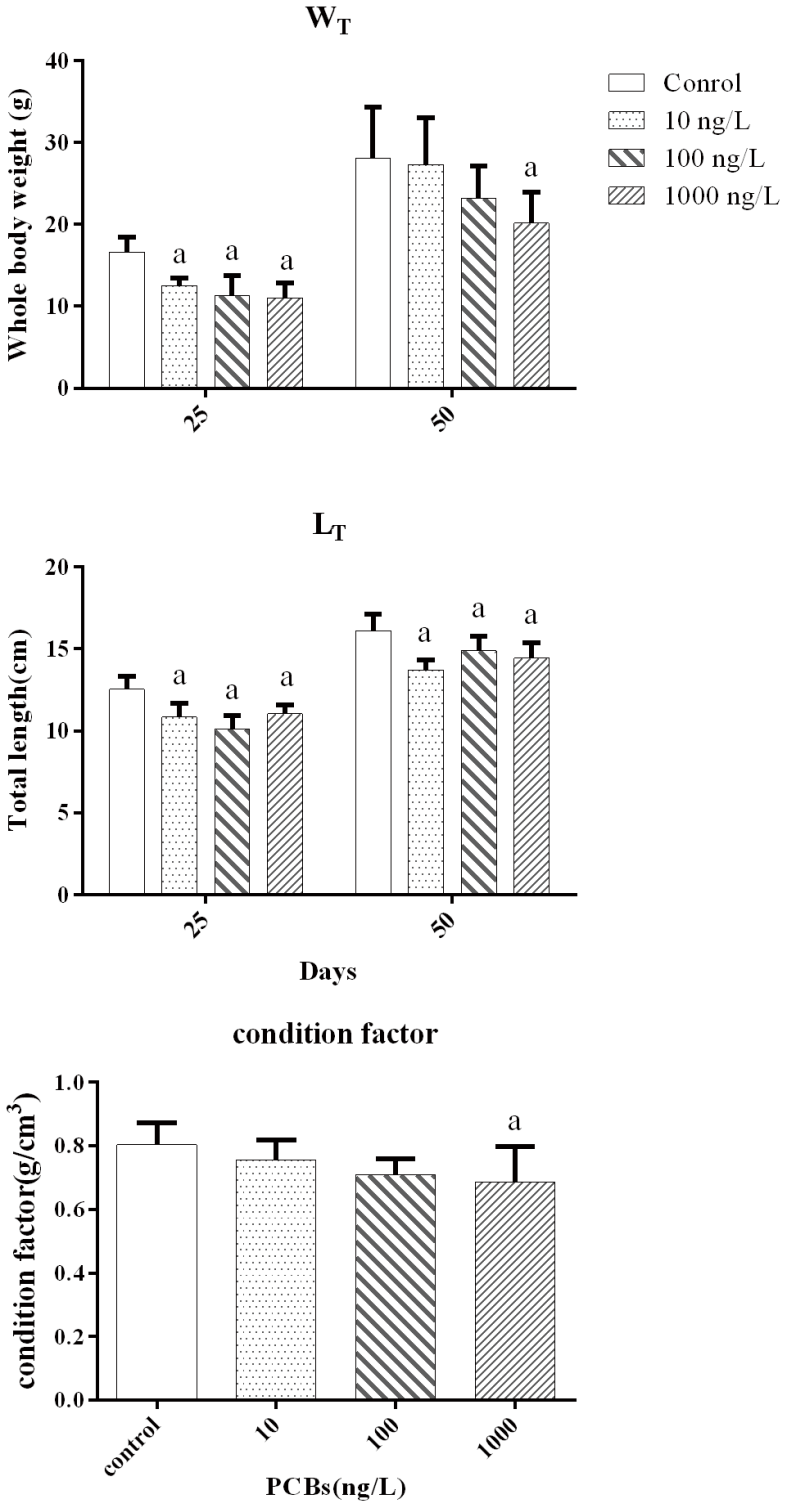
Effect on the total body length, body weight, and condition factor in juvenile Japanese flounder exposed to 0, 10, 100, and 1000/L Aroclor 1254 for 25 and 50 d. The condition factor was calculated at the end of 50^a^ *P*<0.05 indicates significant differences between the exposure groups and corresponding control.

During exposure, mortality rates were below 10% in all groups. As shown in Fig. 1, after 25 days of exposure, Aroclor 1254 significantly reduced W_T_ and L_T_ in all treatments. After 50 days of exposure, 10 ng/L and 100 ng/L Aroclor 1254 did not affect W_T_, but significantly inhibited L_T_. Exposure to 1000 ng/L Aroclor 1254 for 50 days significantly reduced W_T_, L_T_, and CF, relative to the control.

### Effects of Aroclor 1254 on plasma TT_4_, TT_3_, FT_4_, and FT_3_ levels

**Figure 2:**
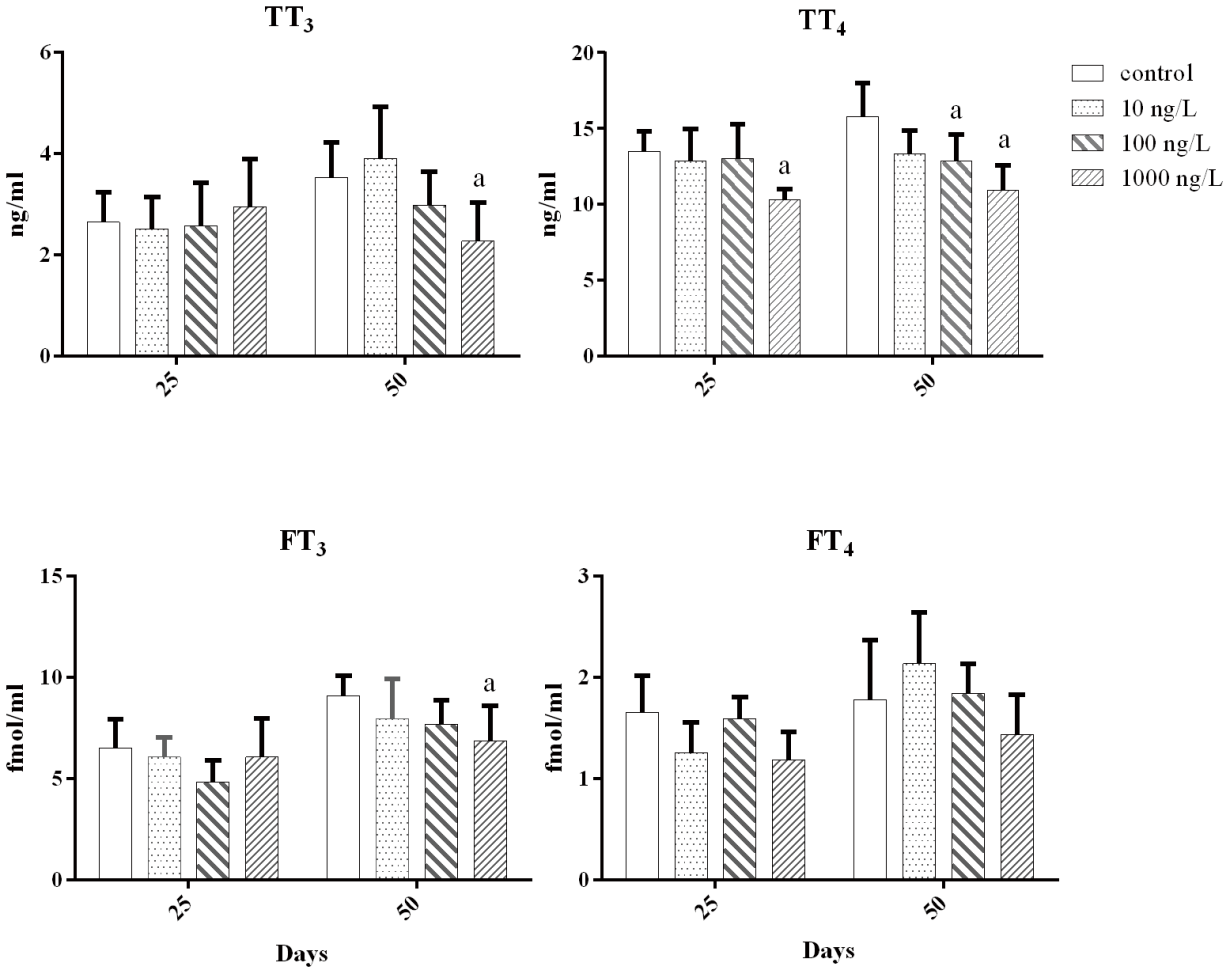
Quantification of plasma TT_3_, TT_4_, FT_3_, and FT_4_ contents in Japanese flounder exposed to 0, 10, 100, and 1000 ng/L of Aroclor 1254 for 25 and 50 d. ^a^ *P*<0.05 indicates significant differences between the exposure groups and corresponding control.

The effects of Aroclor 1254 on plasma THs levels are shown in Fig. 2. In flounder exposed to different concentrations of Aroclor 1254 for 25 days, the TT_3_, FT_3_, and FT_4_ levels in the plasma were not significantly altered by any of the treatments, whereas plasma TT_4_ levels significantly decreased in the 1000 ng/L group. After 50 days of Aroclor 1254 exposure, both plasma TT_3_ and FT_3_ levels significantly decreased in the 1000 ng/L group, with plasma TT_4_ levels showing a dose-dependent decrease, which was significant at concentrations of 100 and 1000 ng/L, while plasma FT_4_ levels remained unaltered.

### Effects of Aroclor 1254 on thyroid histopathology

**Figure 3:**
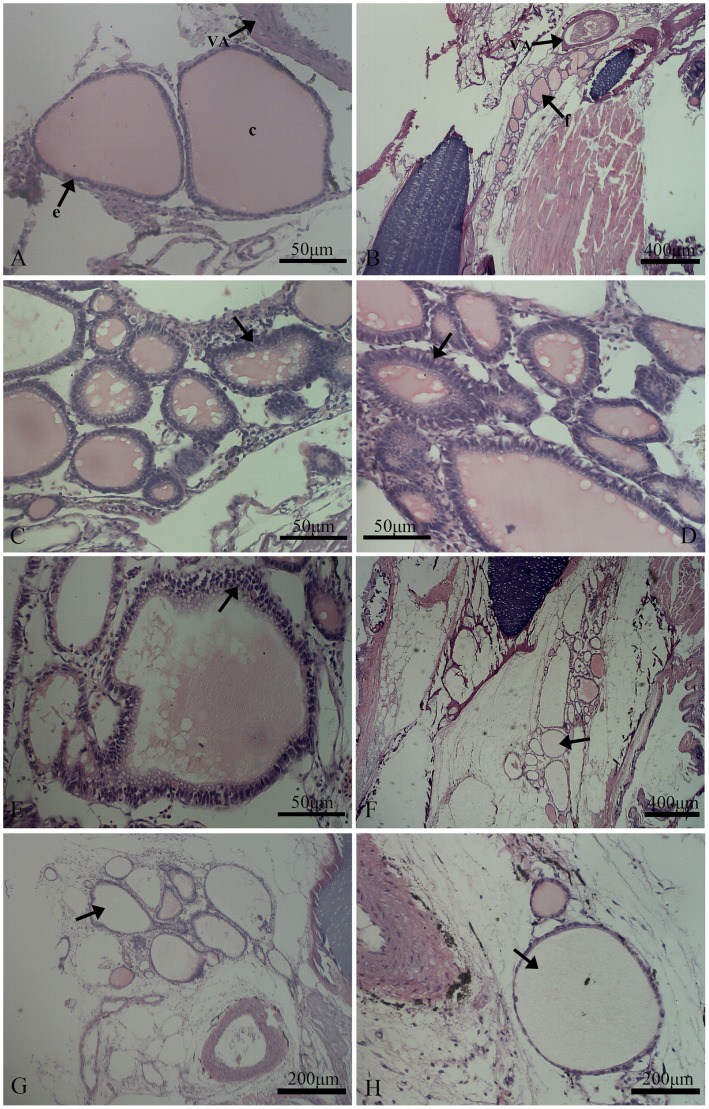
Histological structure of thyroid follicles in juvenile Japanese flounder exposed to 0, 10, 100, and 1000/L Aroclor 1254 for 50 d. (A) and (B) control fish presenting ovoid follicles of variable sizes filled with colloid and lined with squamous follicle cells; (C) and (D) significantly increased epithelial cell height with a little colloid depletion in the lumen after exposure to 100 ng/L Aroclor 1254. (E) Focal hyperplasia in fish exposed to 1000 ng/L. (F) and (G) colloid depletion in fish exposed to 1000 ng/L. (H) Dispersed and reticular colloid in fish exposed to 1000 ng/L. VA = ventral aorta, f = thyroid follicle, c = colloid, and e = thyroid follicle epithelial cell.

**Figure 4:**
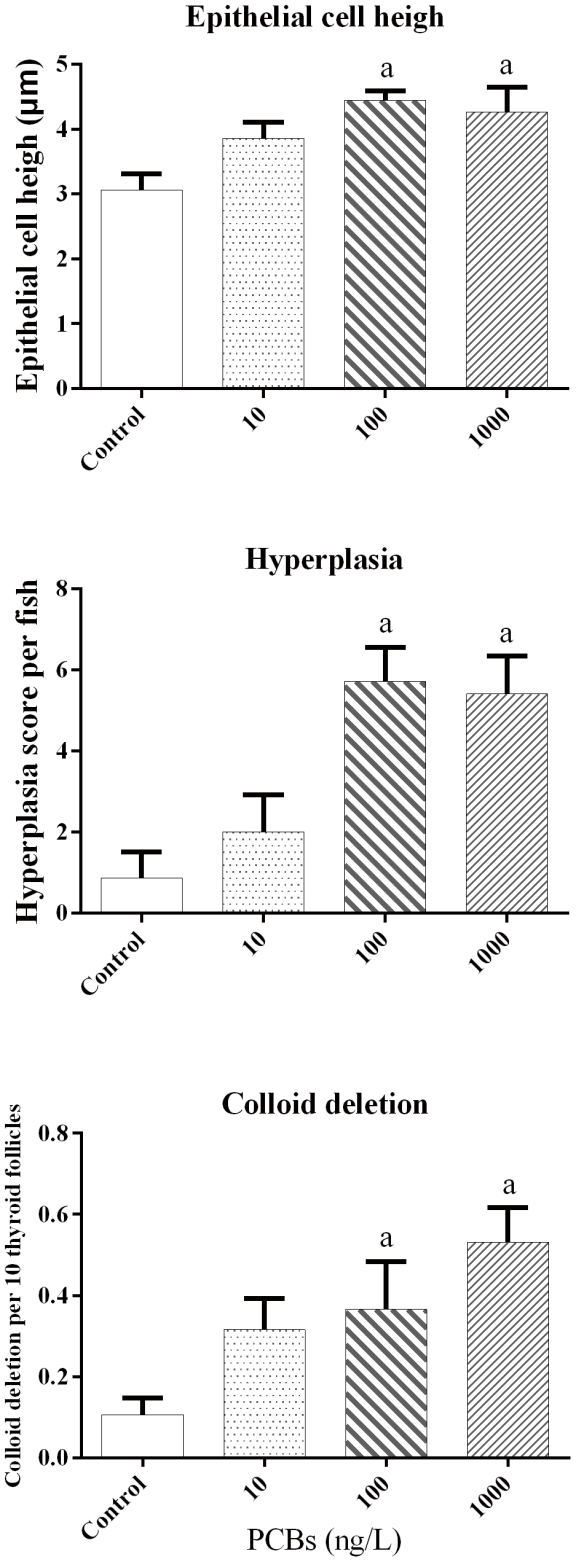
Measurement of epithelial cell height, colloid deletion, and hyperplasia of thyroid follicle in juvenile Japanese flounder exposed to Aroclor 1254 for 50 d. ^a^ *P*<0.05 indicates significant differences between the exposure groups and corresponding control.

The control fish presented oval thyroid follicles of variable sizes that were filled with colloid. In addition, the follicles were line with a single layer of cuboidal to flat follicle epithelial cells (Fig. 3A, B). Representative histopathological abnormalities in Japanese flounder exposed to different concentrations of Aroclor 1254 for 50 days are shown in Fig. 3C–H, including increased epithelial cell height (Fig. 3C, D), hyperplasia (Fig. 3E), and colloid depletion (Fig. 3F, G). Compared to the control group, the colloid observed in the 100 ng/L and 1000 ng/L groups was foamy in appearance, and colloid density decreased (Fig. 3H). For the quantitative analyses, significantly increased levels of follicular epithelial cell height, hyperplasia, and colloid depletion were observed in the 100 and 1000 ng/L Aroclor 1254 treatments (Fig. 4).

### Effects of Aroclor 1254 on ID_1_, ID_2_ and ID_3_ mRNA expression in the liver and kidney

**Figure 5:**
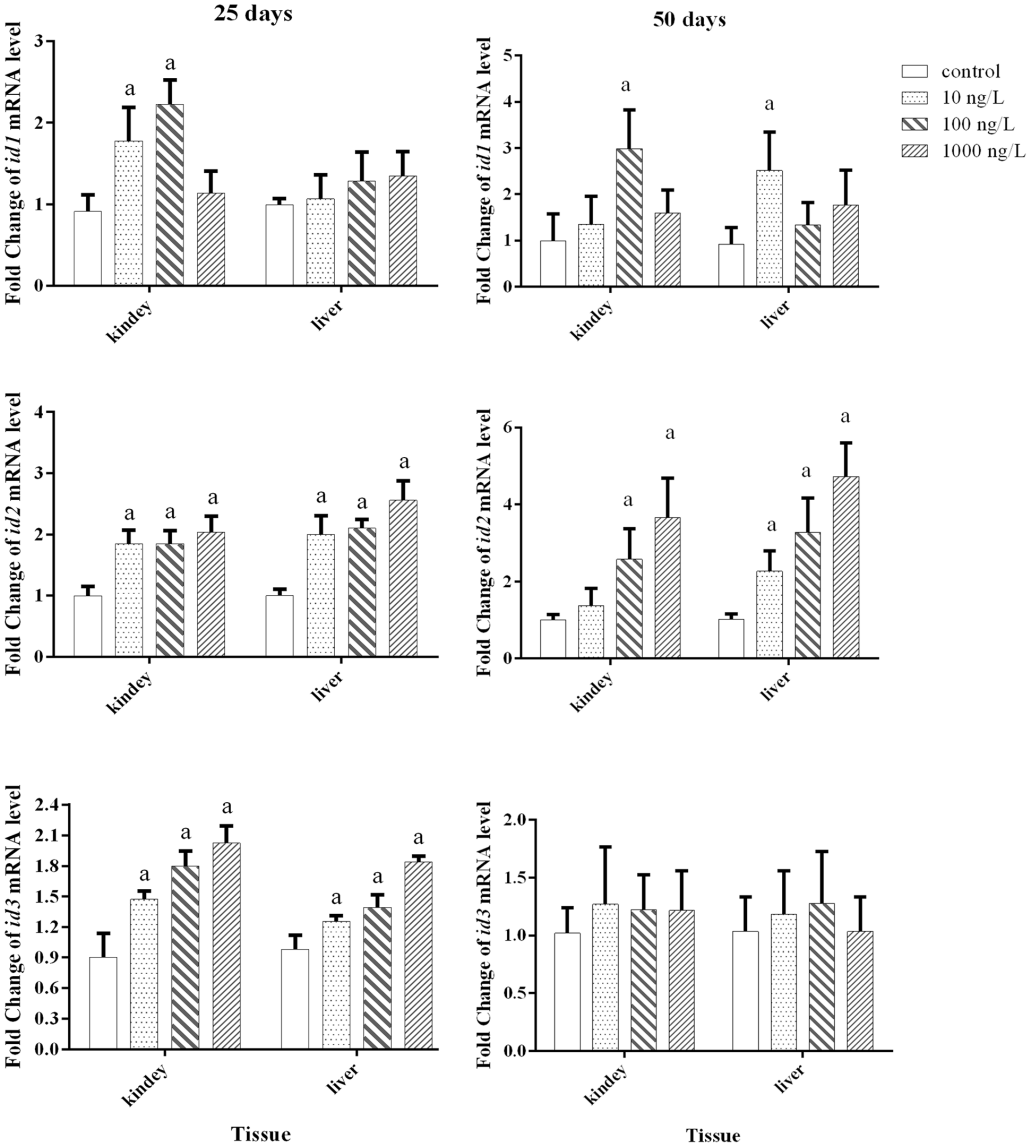
Relative mRNA expression levels of ID_1_, ID_2_, and ID_3_ in the kidney and liver of juvenile Japanese flounder exposed to 0, 10, 100, and 1000 ng/L of Aroclor 1254 for 25 and 50 d. Fold change (y-axis) represents the expression of the target gene mRNA relative to that of the control group (equals 1 by definition). ^a^ *P*<0.05 indicates significant differences between the exposure groups and corresponding control.

As shown in Fig. 5, after 25 days of exposure to Aroclor 1254, ID_1_ mRNA levels in the kidney were significantly higher in the 10 and 100 ng/L grouts; however, no significant difference was observed for the liver in any of the treatments. The significant up-regulation of ID_2_ and ID_3_ mRNA levels was observed in both the kidney and liver of all treatments. In juvenile Japanese flounder exposed to Aroclor 1254 for 50 days, significantly higher ID_1_ mRNA levels were obtained in the kidney and liver of the100 ng/L and 10 ng/L groups, respectively. The transcription of ID_2_ mRNA in the kidney was significantly stimulated on exposure to 100 and 1000 ng/L Aroclor 1254, which were significantly upregulated in the liver for all treatments. The transcription levels of ID_3_ in the kidney and the liver were not significantly altered by any Aroclor 1254 treatment.

## Discussion

Our results showed that exposure to Aroclor 1254 significantly decreased plasma TT_4_ and TT_3_ levels (Fig. 2). However, interpretation of PCBs on the fish thyroid system is exceedingly complex, and does not appear to elicit consistent, detectable plasma TH responses (Table 3). At least three categories of factors have to be considered: 1) test-species variable, 2) the variable composition of PCB mixtures, and 3) the distinction between exposure and effect due in part to thyroid compensation [5].

**Table 3:** The effects of PCBs on fish plasma thyroid hormone homostasis.

Type of PCBs	Dose	Exposure days	Species	Ages of fish	T_4_	T_3_	Reference
PCB 126	25 µg/kg	210	*Salvelinus namaycush*	Adult	**↑**	**–**	[34]
PCB 126	500 µg/kg	7	*Hippoglossoides platessoides*	Adult	**–**	**–**	[44]
PCB 77	1000 µg/kg	90	*Thymallus arcticus*	Adult	**–**	**–**	[49]
PCB 77	500 µg/kg	7	*Hippoglossoides platessoides*	Adult	**↑**	**–**	[44]
Clophen A50	500 mg/kg	10	*Platichthys flesus*	Adult	**–**	**–**	[50]
Aroclor 1254	150 µg/kg	42	*Oncorhynchus kisutch*	Adult	**↓**	**↑**	[6]
Aroclor 1254	1 mg/kg	30	*Micropogonias undulatus*	Adult	**↑**	**↓**	[51]
Aroclor 1254	0.5 µg/g	35	*Oreochromis niloticus*	Adult	**–**	**–**	[38]
Aroclor 1248	5 mg/kg	21	*Ameiurus nebulosus*	Adult	**–**	**↓**	[33]
1254∶1248	50 µg/g	84	*Oncorhynchus kisutch*	Adult	**–**	**↓**	[5]
1254∶1248	10 µg/g	120	*Dicentrarchus labrax*	Adult	**↓**	**↓**	[30]

↑, increase; ↓, decrease; –, no effect.

In this study, Aroclor 1254 exposure inhibited the L_T_, W_T_, and CF of juvenile Japanese flounder, which probably led to growth retardation. Crane et al. [15] found that ammonium perchlorate reduces plasma T_4_ levels, which inhibited the development of fathead minnow (*Pimephales promelas*) larvae. Schmidt et al. [18] reported that exposure of zebrafish larvae to potassium-perchlorate caused a significant decrease in both plasma T_4_ levels and CF. The current study also found that Aroclor 1254 exposure causes plasma T_3_ and T_4_ levels to decline. Because THs are important in the development and growth of teleosts, particularly during the early life stages, this type of thyroid disruption might inhibit the growth of juvenile Japanese flounder.

However, exposure to PCBs produced different results in adult and juvenile fish. For instance, the study by Schnitzler et al. [29] showed that one PCB mixture induced muscle T_4_ levels to decrease in adult sea bass (*Dicentrarchus labrax*), without affecting body length, body weight, or specific growth rates. Iwanowicz et al. [33] reported that the intraperitoneal (*i. p.*) injection of 5 mg/kg Aroclor 1248 caused plasma T_3_ levels to decrease in the brown bullhead (*Ameiurus nebulosus*), but had no significant effects on plasma T_4_ levels or CF. Following exposure to PCB 126 by *i. p.* injection lower plasma T_4_ concentrations was observed in adult lake trout (*Salvelinus namaycush*), whereas it had no effect on fish growth or condition [34]. In adult fish, abundant stores of THs have been found in muscles and other tissues, in addition to the plasma pool, thyroid tissues [35]. These TH stores in extra-thyroidal tissues might be released into the bloodstream or peripheral tissues to compensate thyroid disruption induced by exposure to exogenous compounds. Brown et al. [36] found that muscle T_3_ and T_4_ contents rapidly reduced in rainbow trout exposed to the PCB 126, with few changes in the histology of thyroid follicles and growth rate. This finding indicates that adult fish have a mechanism to compensate for the thyroid system, enabling them to balance available TH content in peripheral tissues, which does not affect growth. In contrast, the peripheral tissues of juveniles contained relatively low TH levels; therefore, TH deficiency in juveniles might be more likely to trigger a negative feedback regulation compared to adult fish, inducing a series of cascading effects that involve the hypothalamus-pituitary-thyroid (HPT) axis to maintain TH homeostasis. Thus, thyroid tissue might stimulate TH synthesis in juvenile Japanese flounder exposed to Aroclor 1254, based on the observed increase in epithelial cell height, hyperplasia of thyroid follicular epithelial cells, and colloid deletion in the current study. This phenomenon might, to some extent, be attributed to the feedback response to Aroclor 1254 within the thyroid cascade.

The severity of colloid depletion and epithelial cell height are routinely employed markers for identifying thyroid disruption [16]. Crane et al. [15] pointed out that colloid depletion indicates serious injuries, close to the collapse of follicles. In the present study, juvenile Japanese flounder exposed to 100 and 1000 ng/L Aroclor 1254 had significantly greater thyroid follicular epithelial cell height, which reduced colloid area. Many irregularly shaped follicles, some without colloids, were observed, particularly in the highest exposure group. These degenerative changes of the thyroid tissues might cause hypothyroidism in juvenile Japanese flounder, preventing them from balance the decrease in TT_4_ baselines in the 2 Aroclor groups with the highest concentrations (100 and 1000 ng/L) after 50 d exposure.

In particular, changes in thyroid tissue histology caused by Aroclor 1254 exposure were similar to those induced by perchlorate. Perchlorate blocks the iodine uptake of thyroid follicles by competitively inhibiting iodide and sodium/iodine transport proteins from combining; thereby, hindering the synthesis of THs [37]. Consequently, the decline in TH levels might stimulate TSH secretion from the pituitary through the feedback pathway, and eventually cause compensatory hypertrophy, hyperplasia, and colloid reduction of thyroid follicular cells [14], [16], [18]. In contrast, some inorganic chemicals, like Cd^2+^, directly damage thyroid follicles by inducing lipid peroxidation; thus, affecting TH synthesis. Therefore, the toxicity mechanism of Aroclor 1254 on thyroid follicles might be similar to that of perchlorate, rather than the direct effect of heavy metals, such as Cd^2+^. In other words, Aroclor 1254 probably causes plasma TH levels to decrease in juvenile Japanese flounder; thereby, inducing the compensatory hypertrophy and hyperplasia of thyroid follicular cells through negative feedback pathways, to promote TH synthesis.

Previous studies have shown that deiodinase in fish is sensitive to environmental contaminants, such as metals, polychlorinated biphenyls, and pesticides [38]–[40]. Van der Geyten et al. [41] demonstrated that changes in hepatic ID_1_ and ID_2_ activities tend to be consistent with that of their mRNA levels, indicating pre-translated regulation, by which deiodinase mRNA levels coincide with deiodinase enzyme activities. In addition, Picard-Aitken et al. [42] suggested that deiodination gene expression could be used as sensitive biomarkers to indicate thyroid disruption in fish on exposure to environmental chemicals. In the present study, the gene expression of IDs in juvenile Japanese flounder was sensitive to exposure to Aroclor 1254. After 25 and 50 d exposure, Aroclor 1254 stimulated the transcription of ID_2_ mRNA in the kidney and liver, which would result in more T_4_ being converted into T_3_. Another study also found that exposure of sea bass to a mixture of Aroclor 1254 and 1248 led to a significant increase in ID_2_ activities [29]. ID_2_ mRNA expression tended to be the most sensitive and stable indicator for thyroid disruption in the present study, because it showed a dose-dependent increase in all treatment groups after both 25 and 50 days exposure, especially in the liver. However, it is difficult to distinguish whether Aroclor 1254 has a direct or indirect disrupting effect on the thyroid system by triggering compensatory mechanisms within the thyroid system; consequently, it is difficult to explain how the thyroid status of juvenile Japanese flounder exposed to Aroclor 1254 is altered by only a few indicators. Blanton and Specker [8] suggested that the actions of certain xenobiotics at different levels of the fish thyroid cascade could not be independently monitored by any biomarker. However, ID_2_ represents one important indicator for interpreting disruption to the thyroid cascade in fish exposed to environmental contaminants.

After 25 d exposure, 10 and 100 ng/L Aroclor 1254 caused ID_1_ and ID_3_ mRNA expression levels to increase, especially in the kidney. This response would accelerate the metabolism of T_3_, which helps maintain plasma THs homeostasis. At the highest dose, the mRNA expression of ID_2_ in the kidney and liver was significantly upregulated, while the expression of ID_1_ showed no significant change. This result also indicates the presence of a compensatory response to decreased plasma TT_4_ levels, to maintain stable plasma TT_3_ levels; otherwise, the increased mRNA expression of ID_3_ in the kidney and liver might aggravate the reduction in plasma TT_4_. In studies of tilapia, van der Geyten et al. [43] found that ID_2_ activity in the liver and ID_3_ activity in the gill decreased with declining T_3_ concentrations, which is responsible for balancing the reduction in T_3_. Schnitzler et al. [29] suggested that PCB-induced changes in deiodinase activity offset the decline in plasma T_3_ levels. Adams et al. [44] suggested that elevated T_4_ ORD activity serves as a homeostatic adjustment to offset increased systemic T_3_ clearance. After 50 d exposure, the decrease in plasma TT_3_ levels at the highest dose was mostly due to hypothyroidism, which caused a drop in thyroidal T_4_ production and secretion; thus, exceeding the regulation ability of IDs, and also resulting in lower FT_3_ levels.

Some authors have found that a change in plasma TH levels alters the ID_3_ expression. For example, Higgs and Eales [45] found that a decrease in fish T_4_ levels leads to a decrease in the metabolic clearance level of T_4_; in other words, a decrease in the ID_3_ level. A study by Van der Geyten et al. [41] showed that a decrease in the TT_4_ and TT_3_ levels of tilapia with thyroid dysfunction caused by methimazole exposure caused ID_1_ and ID_2_ levels to increase and ID_3_ levels to decrease. However, the current study found that a decrease in plasma TH levels did not influence the mRNA expression of ID_3_ after 50 days of exposure. Coimbra et al. [38] found that at 21 and 35 days after tilapia were exposed to Aroclor 1254, TT_3_ and TT_4_ levels showed no significant changes, whereas ID_3_ activity levels in the liver significantly increased, while the activity of ID3 increased in the gill after 21 days of exposure. The exact reason for this phenomenon requires further study.

At present, higher exposure concentrations of PCBs are often used to investigate their thyroid-disrupting effects on adult fish (Table 3). Of note, PCB concentrations detected in the environment are far lower than those used in these exposure experiments. For example, the PCBs content of the surface water and sediment in the Minjiang River Estuary, China, are 985 ng/L and 34.39 µg/kg on average, respectively [46]. The total concentration of PCBs ranged from 2.33 µg/kg to 44 µg/kg in the marine sediments in Barcelona, Spain [47], and 10 µg/kg to 899 µg/kg in the surface sediments of Naples Harbour, Italy [48]. Adult sea bass fed with the equivalent of actual environmental concentrations of the mixture of Aroclor 1260 and 1254 only showed reduced muscle T_3_ levels, with no significant changes in muscle T_4_ levels and thyroid histology; however, exposure to the same contaminants at concentrations 10 times above actual environmental concentrations led to a decrease in both T_3_ and T_4_ levels in muscles, and caused follicular degeneration [29]. This study found that even environmentally relevant concentrations of Aroclor 1254 caused significant disruption to the thyroid system of flounder juveniles, including changes in thyroid histopathology, altered plasma TH levels, and modulation in the expression levels of IDs mRNA in the liver and kidney. This result supported the hypothesis that juvenile fish are more sensitive to PCBs compared to adult fish, making them suitable candidate animal models for studying TDCs.

Many TDCs, such as sodium perchlorate, have been reported to affect the early growth and development of teleosts [15], [16], [18]. Mechanisms underlying the effects of PCBs on the early life stages of fish development *via* their thyroid disrupting abilities should be investigated in future studies, not only to delineate the disrupting effects of PCBs at individual and ecological levels, but also to establish some links between the macroscopic effects and the microscopic mechanisms for a more comprehensive ecological risk assessment of these pollutants. In particular, flatfish species, including Japanese flounder, experience a unique and critical process of metamorphosis during development, when the larvae shift from a planktonic to a benthic mode of life, with this process being primarily controlled by the thyroid system. Therefore, the larvae of Japanese flounder may represent an excellent model organism for investigating the effects of PCBs on the thyroid system and fish development in future studies.
